# Outbreak of *Ichthyophthirius multifiliis* associated with *Aeromonas hydrophila* in *Pangasianodon hypophthalmus*: The role of turmeric oil in enhancing immunity and inducing resistance against co-infection

**DOI:** 10.3389/fimmu.2022.956478

**Published:** 2022-09-02

**Authors:** V. Kumar, B. K. Das, H. S. Swain, H. Chowdhury, S. Roy, A. K. Bera, R. Das, S. N. Parida, S. Dhar, A. K. Jana, B. K. Behera

**Affiliations:** ^1^ Aquatic Environmental Biotechnology and Nanotechnology (AEBN) Division, Indian Council of Agricultural Research (ICAR)-Central Inland Fisheries Research Institute (CIFRI), Barrackpore, India; ^2^ Indian Council of Agricultural Research (ICAR)-Central Inland Fisheries Research Institute (CIFRI), Barrackpore, India; ^3^ Fisheries Enhancement and Management (FEM) Division, Indian Council of Agricultural Research (ICAR)-Central Inland Fisheries Research Institute (CIFRI), Barrackpore, India; ^4^ Reservoir and Wetland Fisheries (RWF) Division, Indian Council of Agricultural Research (ICAR)-Central Inland Fisheries Research Institute (CIFRI), Barrackpore, India; ^5^ Fisheries Resource Assessment and Informatics (FRAI) Division, Indian Council of Agricultural Research (ICAR)-Central Inland Fisheries Research Institute (CIFRI), Barrackpore, India

**Keywords:** *I. multifiliis*, *A. hydrophila*, *P. hypophthalmus*, turmeric oil, anti-stress, antioxidative response, immunity

## Abstract

*Ichthyophthirius multifiliis*, a ciliated parasite causing ichthyophthiriasis (white spot disease) in freshwater fishes, results in significant economic loss to the aquaculture sector. One of the important predisposing factors for ichthyophthiriasis is low water temperature (i.e., below 20°C), which affects the health and makes freshwater fishes more susceptible to parasitic infections. During ichthyophthiriasis, fishes are stressed and acute immune reactions are compromised, which enables the aquatic bacterial pathogens to simultaneously infect the host and increase the severity of disease. In the present work, we aimed to understand the parasite–bacteria co-infection mechanism in fish. Later, *Curcuma longa* (turmeric) essential oil was used as a promising management strategy to improve immunity and control co-infections in fish. A natural outbreak of *I. multifiliis* was reported (validated by 16S rRNA PCR and sequencing method) in *Pangasianodon hypophthalmus* from a culture facility of ICAR-CIFRI, India. The fish showed clinical signs including hemorrhage, ulcer, discoloration, and redness in the body surface. Further microbiological analysis revealed that *Aeromonas hydrophila* was associated (validated by 16S rRNA PCR and sequencing method) with the infection and mortality of *P. hypophthalmus*, confirmed by hemolysin and survival assay. This created a scenario of co-infections, where both infectious agents are active together, causing ichthyophthiriasis and motile *Aeromonas* septicemia (MAS) in *P. hypophthalmus*. Interestingly, turmeric oil supplementation induced protective immunity in *P. hypophthalmus* against the co-infection condition. The study showed that *P. hypophthalmus* fingerlings supplemented with turmeric oil, at an optimum concentration (10 ppm), exhibited significantly increased survival against co-infection. The optimum concentration induced anti-stress and antioxidative response in fingerlings, marked by a significant decrease in cortisol and elevated levels of superoxide dismutase (SOD) and catalase (CAT) in treated animals as compared with the controls. Furthermore, the study indicated that supplementation of turmeric oil increases both non-specific and specific immune response, and significantly higher values of immune genes (interleukin-1β, transferrin, and C3), HSP70, HSP90, and IgM were observed in *P. hypophthalmus* treatment groups. Our findings suggest that *C. longa* (turmeric) oil modulates stress, antioxidant, and immunological responses, probably contributing to enhanced protection in *P. hypophthalmus*. Hence, the application of turmeric oil treatment in aquaculture might become a management strategy to control co-infections in fishes. However, this hypothesis needs further validation.

## Introduction

*Ichthyophthirius multifiliis*, a holotrichous protozoan ciliated parasite, is the causative agent of ichthyophthiriasis (white spot disease) and a major problem to aquarists and commercial fish producers worldwide (1). The infection can occur at several growth stages starting from day-old fry, fingerling, and food size to brood fish. The infective stage of the parasite, the theront, invades gills and skin of the fish and penetrates the epidermis layer to settle above the basal lamina (2). In most cases, this may lead to epidemic conditions and high mortalities can be observed in fish, resulting from either theront penetration, parasites feeding on cells and tissues or trophont escape from the fish surfaces (3, 4). Interestingly, during ichthyophthiriasis, a series of pathological events takes place that leads to high morality in fishes. For instance, the parasite damages fish skin/gills and breach fish first line of defense, which helps bacterial pathogens gain entry into fish host and causes stress. This also resulted in reduced fish immune response, thus increasing the ability of bacterial pathogen to infect the fish (5, 6). However, *I. multifiliis* causing mass mortalities and morbidities in freshwater fishes are often associated with co-infections condition, which most likely involve two or more pathogens. The co-infection studies involving bacteria–bacteria (e.g., *Aeromonas* and *Flavobacterium*) is well defined in fishes; however, bacteria–parasitic co-infection is very poorly delineated. Shoemaker etal. (7) reported that severity of disease outbreak and mortality increased in *I. multifiliis*-infected moribund fish when co-infected with bacterial pathogens (7).

The *Aeromonas hydrophila* is a Gram-negative, facultative anaerobic, motile bacterium that is the causative agent of motile *Aeromonas* septicemia (MAS). This bacterium is widely distributed in aquaculture and can cause significant losses in the presence of a predisposing stressor (8). The MAS in fish has two forms: acute hemorrhagic septicemias characterized by generalized edema, hemorrhage, and diffuse necrosis; and chronic ulcerative syndrome marked by the formation of deep dermal ulcers (9–11). MAS outbreaks in the aquaculture system are seasonal, with peaks generally occurring in the spring to early summer and fall when the water temperatures are between 18 and 29°C (12). Interestingly, the temperature ranges for MAS outbreaks overlap with the optimum temperature window of ichthyophthiriasis infection at 18 to 27°C (3). In parallel, there are few reports that suggest that severity of parasitism by *I. multifiliis* could be enhanced by *A. hydrophila* infection resulting in higher mortality of fish species (13). Hence, there is a need to understand the co-infection mechanism in fishes in order to develop a suitable management method to control the parasite and bacterial co-infection and reduce disease outbreak and mortality.

The traditional methods applied so far in the mitigation or cure of parasitic and bacterial infection such as disinfectants and antibiotics had very limited success (14). Additionally, their application in the food-production sector is under severe public and scientific scrutiny due to the development of multiple antibiotic resistances in microbial phenotypes (15, 16). This created a huge concern among food production and drug development regulatory bodies, and more emphasis has been given in the development of plant-based and/natural compounds to enhance the immune reactivity and disease resistance in fishes against pathogenic microbes (17). The use of plant-based compounds or natural products has attracted keen interest in the past decades due to their antimicrobial, immunostimulant, and antioxidant properties. Harikrishnan etal. (18, 19) demonstrated that dietary supplementation of plant-based compounds significantly improves immune response and disease resistance in fishes infected with microbial pathogen. Afzali and Wong (20) reported that dietary supplementation of *Sonneratia alba* extract enhances immunity and provides protection and disease resistance in goldfish. Kumar etal. (21, 22) further highlighted that plant-based compounds (*Mikania cordata* leaf powder) can be used as a potential immunostimulatory agent to enhance non-specific immune response and disease resistance of fishes. Recently, Sheikhzadeh etal. (23) reported that dietary sodium propionate enhanced the growth performance, immune-related genes expression, and resistance of goldfish (*Carassius auratus*) against *I. multifiliis* (23). Although these studies have shown promising results in enhancing immune reactivity against pathogenic microbes, still no work has been done to control the disease outbreak and mortality in fishes during co-infection.

*Curcuma longa* (turmeric), a functional herbal spicy plant affiliated with the family Zingiberaceae, has several ethnomedicinal properties. Apart from protein, lipid, mineral, and carbohydrate content, turmeric contains curcuminoids, which are a mixture of curcumin (diferuloylmethane), monodesmethoxy curcumin, and bis desmethoxy curcumin (24, 25). Markedly, curcuminoids have antibacterial, growth-promoting, immunostimulant, anti-inflammation, and antioxidative properties in animals and humans (26). In aquaculture, dietary supplementation of curcumin has been shown to enhance growth, immunity, antioxidative status, and disease resistance in finfish species (27, 28). However, the potential role of curcumin has not been evaluated against co-infection conditions. In this study, we aimed to characterize a disease condition that indicated the possible involvement of multiple pathogens, a condition known as co-infection. Furthermore, experiments were designed to investigate whether turmeric oil potentiates generation of anti-stress, antioxidant, non-specific, and specific immunity in *Pangasianodon hypophthalmus* against co-infection. Subsequently, it was verified whether this immunostimulatory role could contribute to the generation of protective response against *I. multifiliis* and *A. hydrophila* co-infection.

## Materials and methods

### Outbreak description

In mid-December 2021 to end of January 2022, we investigated a case of severe mortality, >90% (270 out of 300), in fingerlings of *P. hypophthalmus* (length = 140.4 ± 5.3 mm, weight = 15.83 ± 2.8 g) kept at a culture facility of ICAR-CIFRI, Barrackpore, India. Based on the available records, the water quality parameters (except temperature) were maintained following standard guidelines. Interestingly, the water temperatures in the culture tanks during the outbreak period (ranging from 16 to 20°C) were below the optimum fish growth temperature (~28°C). The fish were showing signs of disease, including extreme lethargy, presence of white spot, hemorrhage, ulcer, discoloration, and redness in fins and all over the body surface. Suspecting a microbial infection, moribund fingerlings of *P. hypophthalmus* showing clinical signs were transported to the laboratory, necropsied, and subjected to further microbiological tests.

### Parasite isolation and identification by 18S rRNA gene and phylogenetic analysis

In laboratory, fresh smears of skin and gills were examined under a microscope to observe the possible presence of parasites. The *I. multifiliis* tomonts were collected from heavily infected *P. hypophthalmus* (~20) using standard protocol (29). The slow-swimming tomont stages were individually isolated and transferred to a 2-ml microcentrifuge tube for DNA isolation. DNA from tomonts was prepared by lysing the cells in 1% sodium dodecyl sulfate, 0.5 M EDTA, and 10 mM Tris (pH 9.5) for 20 min at 65°C followed by incubation in 0.5 mg/ml pronase in a mixture of 0.6% sodium dodecyl sulfate, 0.5 M EDTA, and 10 mM Tris (pH 9.5) for 18 h at 56°C. DNA was isolated by the phenol-chloroform extraction method, precipitated with ethanol, and resuspended in 1 mM Tris–10 mM EDTA (pH 8.0) (30).

PCR amplification of parasitic nucleotide was performed using universal primers (**Table 1**) that target ‘‘highly conserved 18S rRNA gene V4 regions’’ (31). The extracted DNA quality was checked on agarose gel (1%) and quantified using Nano-drop (Eppendorf, Germany). The 18S rRNA gene was amplified using a Gene Amp PCR system 9700 thermal cycler (Applied Biosystems, Foster City, CA). A total of 50 μl of the PCR reaction mixture consists of 5 μl of 10× PCR buffer, 1 μl of 10 mM dNTP, 1 μl of 50 mM MgCl_2_, 1 μl of 10 pmol of each primer, 1 U Taq DNA polymerase, and 100 ng of isolated genomic DNA. The PCR condition includes 5 min at 94°C initial denaturation, 35 cycles of 94°C for 30 s (denaturation), 54°C for 30 s (annealing), 72°C for 60 s (extension), and 5 min at 72°C of final extension. Amplified products were visualized on 1.8% agarose gel (32) (**Table 1**). The amplified gene was sequenced in forward and reverse direction using ABI 373xl capillary sequencer (Applied Biosystem, Foster City, CA). Contig was prepared by aligning forward and reverse sequence using DNA baser 7.0.0. The sequence was submitted to GenBank and phylogenetic tree was constructed using the Neighbor-Joining method in MEGA X.

**Table 1:** List of primers used for amplification of the standardized 16S and 18S rRNA gene.

Target gene	Primers	Sequence (5’-3’)	Size of amplicon (bp)
16S rRNA	16S-F	GTTGATCATGGCTCAG	1,414
	16s-R	GGTTCACTTGTTACGACTT	
18S rRNA	18S-F	ACCTGGTTGATCCTGCCAG	1,700
	18s-R	CTTCCGCAGGTTCACCTACGG	

### Bacterial isolation and identification by 16S rRNA gene and phylogenetic analysis

The infected fish (~20) with the distinct clinical sign were anesthetized using clove oil (Merck, Germany) at 50 μl/L dose. Under axenic condition, kidney, liver, and blood samples were collected and aseptically homogenized in phosphate buffer saline (PBS). The 200-μl samples were transferred onto tryptic soya agar plates (TSA) (Himedia, India) and incubated at 37°C for 24 h. The growth of morphologically similar colonies on TSA plates indicates the presence of a single bacterial strain. A single colony was streaked onto fresh prepared TSA plates and incubated at 37°C for 24 h to obtain a pure strain. Later, the single colony was streaked into thiosulfate citrate bile salt sucrose agar (TCBS) plates and incubated at 37°C for 24 h. Subsequently, the colony was transferred to tryptic soy broth (TSB), and after 24 h of incubation at 37°C, the bacterial strain stock culture was prepared in 30% glycerol and stored at −20°C until further used. Later, the target bacterial strain was enriched with alkaline peptone water (APW) and hemolysin, biochemical test, antibiotic susceptibility test, challenge experiment, and DNA isolation were performed.

The genomic bacterial DNA was isolated following the standard Sarkosyl method (33). In brief, the quality of DNA extracted was checked on agarose gel (1%) and quantified using Nano-drop (Eppendorf, Germany). The 16S rRNA gene was amplified by Gene Amp PCR system 9700 thermal cycler (Applied Biosystems, Foster City, CA) using universal bacterial primers (**Table 1**). Fifty microliters of the PCR reaction mixture consisted of 10× PCR buffer (5 μl), 50 mM MgCl_2_ (1 μl), 10 mM dNTP (1 μl), 10 pmol of each primer (1 μl), isolated genomic DNA (100 ng), and Taq DNA polymerase (1 U). The PCR condition is composed of 2 min at 95°C initial denaturation, 35 cycles of 94°C for 30 s denaturation, 52°C for 60 s annealing, 72°C for 90 s extension, and 7 min at 72°C of final extension. Subsequently, the PCR-amplified products were visualized on agarose gel (1.8%) (34). The amplified gene was sequenced in forward and reverse direction using ABI 373xl capillary sequencer (Applied Biosystem, Foster City, CA). Contig was prepared by aligning forward and reverse sequence using DNA baser 7.0.0. The sequence was submitted to GenBank and phylogenetic tree was constructed using the Neighbor-Joining method in MEGA X.

### Histopathology

Moribund fish showing clinical signs (~10) were anesthetized with Clove oil (at 50 µl per liter of water) and used for collection of tissue. The post-mortem examination was done to observe and record gross lesions in internal organs of fish. Tissue samples from liver were collected and fixed in neutral buffered formalin (NBF) (10%). The fixed tissues were washed and cut into 1- to 2-mm small pieces. Then, the samples were dehydrated in ethanol with different concentrations followed by treatment with the clearing agent xylene. The cleared tissue was embedded into paraffin using an impregnation technique (Leica EG 1140H, Germany). Sectioning of the paraffin-embedded tissue was done using a microtome, maintaining a thickness of 5 μm, followed by staining with hematoxylin and eosin (35). Afterwards, the sections were observed under a light microscope for cellular and pathological changes.

### Bacteria virulence characterization

#### Ethical approval

Organization for Economic Cooperation and Development (OECD) guidelines were followed for the handling and care of experimental animals. The animal utilization protocol was approved by the Institutional Animal Ethics Committee, ICAR-Central Inland Fisheries Research Institute, Kolkata, India (IAEC/2021/04) for the experimental setup.

#### Fish challenge assay

Healthy *P. hypophthalmus* (*n* = 300; length = 134.2 ± 3.9 mm, weight = 14.2 ± 1.9 g) were purchased from a local fish hatchery. The fish that appeared normal and healthy with no external clinical symptoms like ulcer, hemorrhage, scale loss, discoloration, and redness in the body surface was used in the experiment. Additionally, the fish were randomly selected (*n* = 20) and screened for the possible presence of infectious microbes following the standard protocol (36). Briefly, the fish were screened using virulent gene-specific primers with PCR methods for common freshwater microbial pathogens, including *Aeromonas*, *Pseudomonas*, *Flavobacterium*, and *Edwardsiella* species. Afterwards, acclimatization of fish was done for 2 weeks in 200-L fiber-reinforced plastic (FRP) tanks, supplied with proper commercial floating feed (crude protein: 30%, crude lipid: 5%) with 3%–5% of body weight fed twice a day. During the culture period, the photoperiod was maintained with a regimen of 12-h light and 12-h darkness, provided with aeration (DO 6.8–7.2), and the water temperature was maintained at 27.5–28.5°C in a controlled temperature room. Subsequently, the bacterial strain was cultured in 20 ml of sterile TSB in a 50-ml Erlenmeyer flask (Himedia, India) for 24 h at 37°C. Bacterial cell pellets were collected by centrifuging for 5 min at 5,000 rpm and washed thrice with a sterile normal saline solution. Afterwards, the pellets were resuspended in saline solution and the number of cells (CFU/ml) was estimated through the spread plate method. The experimental fish were intraperitoneally injected (20/concentration) with 200 µl (10^2^, 10^3^, 10^4^, 10^5^, 10^6^, and 10^7^ colony-forming units/ml) of bacterial suspension. Control fish were injected with 200 µl of sterile saline solution. Afterwards, the fish were kept in an FRP tank and observed every 24 h until 120 h. To confirm Koch’s postulate, the bacteria were reisolated and identified from the liver, kidney, and blood of the moribund fish.

#### Hemolysin assay

The hemolytic activity of the bacterial strain was conducted according to standard protocol with slight modifications (16). Briefly, the tryptone soya agar (TSA) plates were prepared by supplementing 5% defibrinated sheep blood. The pure stock cultures of bacterial strain were grown overnight in TSB at 37°C under constant agitation. Later, the overnight culture was diluted to 0.5 (OD_600_) and 2 µl of the diluted culture was spotted in the middle of the hemolysin test plates. The plates were incubated at 37°C and diameters of the clearing zones were measured after 48 h. The assay was performed in five replicates with freshly prepared media.

### Extraction of *C. longa* (turmeric) oil

The rhizomes of *C. longa* L. (turmeric) TCP-2 variety were collected from the local market of the North Bengal part of West Bengal, India. The rhizomes were grown for two years in the institute research farm of ICAR-CIFRI, Barrackpore, West Bengal, India. The farming area, situated at longitude 22°C45’N and latitude 88°C26’E, has a warm and humid climate, and constitutes of Gangetic alluvial soil with neutral pH and good fertility status recording an average annual rainfall of 1,350–1,500 mm. Plants were grown in raised beds with moist and well-drained loose soil maintaining standard interspace, during May–January, and recommended agronomic practices were followed to grow the crop. Matured rhizomes with a length of 5–10 cm and a diameter of 1–3.5 cm were selected for extraction of oil. Fresh rhizomes were washed in running water, shade dried for half an hour, sliced to thin pieces, and then extracted in a Clevenger apparatus for 3 h. Extracted oil, yield of 2.65% (V/W), was collected and dried over sodium sulfate bed and then stored in an amber-colored bottle at 4°C for further use in the experiment.

### Analysis of chemical composition of the turmeric oil

The essential oil was analyzed by GC-MS (7890A GC, Agilent Co., USA), and its chemical composition was determined using an HP-5MS column (30 m × 0.25 mm; 0.25 µm; Agilent Co., USA). The instrument is directly connected to a triple-axis HED-EM 5975C Mass Spectrometer (Agilent Co., USA) with a split control inflow mode enabled with 1 µl of injection volume. Helium (high purity, New Delhi, India) with a head pressure of 10 psi is used as the carrier gas with a flow rate of 1 ml min^−1^. The oven temperature was initially raised to 60˚C for 1 min and gradually increased to the interface temperature of 250˚C maintaining a gradient of 4˚C min^−1^. The ion source temperature was retained at 200˚C and electron impact ionization (EI) was performed at 70 eV full scan mode and selected ion monitoring (SIM) mode was implemented for mass spectra analysis (37, 38).

The MSD productivity Chemstation program was used to obtain the pure spectrum by processing the raw MS data and excluding the residual background contaminants, column bleed, and partially eluted peaks (**Figure S1**). The interface temperature at 280˚C, ion source temperature at 200˚C, electron ionization at 70 eV, full scan mode of 50–550 mass units, solvent delay by 3 min, and EM voltage of 889 were functional for MS acquisition (**Table S1**). The chemical structures were concluded from the inbuilt library in the instrument.

### *C. longa* (turmeric) oil lethality test and challenge assay

#### Collection of experimental fish

To conduct the assay, we used infected (length = 140.4 ± 5.3 mm and weight = 15.83 ± 2.8 g, collected from the ICAR-CIFRI culture facility) and healthy *P. hypophthalmus* fingerlings (length = 134.2 ± 3.9 mm and weight = 14.2 ± 1.9 g, purchased from a local fish hatchery). As mentioned above in the bacterial fish challenge assay, the healthy fish were screened for any external symptoms and possible infectious microbes following the standard protocol (36). Before the assay, the fish were acclimatized for 2 weeks and fed with commercial diet daily in two equal installments (10:00 h and 15:00 h).

### Lethality test of *C. longa* (turmeric) oil

In the first experiment, the toxic effect of turmeric oil was determined in the healthy *P. hypophthalmus* as described previously by Kumar etal. (39) with slight modifications. Briefly, 20 P*. hypophthalmus* fingerlings were randomly distributed in 200-L FRP tanks containing 100 L of water. The fingerlings were exposed to increasing concentrations of turmeric oil (dissolved in 1% DMSO), viz., 2.5 (T1), 5 (T2), 10 (T3), 20 (T4), 40 (T5), and 80 ppm (T6). The *P. hypophthalmus* fingerlings that have not received turmeric oil served as a control group. The toxicity of the turmeric oil was determined 120 h post-exposure by calculating the number of survived animals as previously described (40). Both treatment and control groups were maintained in five replicates.

### Dose–response study

In the next experiment, the dose–response relationship (protective effect) of turmeric oil was investigated. As described above in the lethality study, 20 P*. hypophthalmus* fingerlings were randomly distributed in 200-L FRP tanks. The tanks were supplemented with increasing concentrations of turmeric oil (non-lethal dose, based on the previous toxicity assay), viz., 2.5 (T1), 5 (T2), 10 (T3), and 20 ppm (T4) (40). Subsequently, the survival of *P. hypophthalmus* was scored 120 h post-turmeric oil addition. The non-exposed fingerlings (turmeric oil) that were either co-infected or not (healthy animals) were maintained as positive and negative controls. The treatment and control groups were maintained in quintuplicate.

### Collection of blood from the fish and separation of serum

Five fish from the control and treatment groups were randomly selected to collect blood samples for biochemical analysis. Briefly, the fish were anesthetized with clove oil (with 50 µl per liter water) and a 2-ml hypodermal syringe was used to collect blood by puncturing the caudal vein of fish. The blood samples collected in sterile Eppendorf tubes, without anticoagulant, were stored overnight at 4°C. Afterwards, the samples were centrifuged at 4,000 × *g* for 10 min at 4°C and straw-colored serum was collected and stored at −20°C, until further used for analysis. All the procedures were carried out in the sterilized condition.

### Serum biochemical analysis

#### Antioxidant enzymes

The activity of antioxidant and metabolic enzymes was measured in serum following the standard method with slight modifications. Superoxide dismutase (SOD) activity was measured according to standard protocol in a medium containing sodium carbonate buffer (pH 10.2), EDTA, enzyme extract, and epinephrine (41). The change in absorbance was monitored at 480 nm using Microplate reader (BioTekEpoch™2 Take-3 plate reader, USA).

The standard method of (42) was used to determine the activity of catalase (CAT) (43). The breakdown of H_2_O_2_ was determined by measuring the intensity at 240 nm. The reaction solution comprised 50 mM H_2_O_2_ and 50 mM phosphate buffer (pH 7.2) and the reaction efficiency was determined by measuring the absorbance at 240 nm using Microplate Reader (BioTekEpoch™2 Take-3 plate reader, USA) calibrated to 320 nm H_2_O_2_ having an extinction coefficient of 40 M^−1^ cm^−1^. The CAT activity is expressed as one unit of H_2_O_2_ decomposed per minute and per milligram of protein.

#### Serum biochemical indices and immune-stress responses

Serum protein was measured by using an automated biochemical analyzer (Auto Analyzer, Transasia-Erba EM–200, USA). The heat shock protein 70 (HSP70) and 90 (HSP70) activity in serum was quantified using standard protocols and reagents provided by the manufacturer. These assays were performed using an ELISA assay kit (BT Bio Assay, Shanghai, China) as per the manufacturer’s instruction, and the final OD was taken at 450 nm using a Microplate reader (BioTekEpoch™2 Take-3 plate reader). The assay is representative of two independent experiments, each performed in triplicate.

For cortisol, a commercial ELISA kit was used for analysis following the manufacturer’s instruction (Bioassay Technology Laboratory, China). Briefly, 20 μl of each cortisol standard solution (0, 20, 50, 100, 200, 400, and 800 ng ml^−1^) and fish serum sample were added in triplicate to the microplate. Similarly, the samples for the recovery and linearity test were dispensed in other wells. Standard solutions and fish serum samples for recovery test were assayed in duplicate. Subsequently, 200 μl of enzyme conjugated to horseradish peroxidase was added into each well. Later, the wells were gently mixed for 10 min and incubated for 1 h at room temperature. The solution of each well was removed by washing the plate three times with 400 μl of PBS and shaking out the content onto absorbent paper with the aim of removing residual drops that could affect the accuracy and precision of the assay. Subsequently, 100 μl of TMB (tetramethyl benzidine) enzyme substrate was added to each well and incubated for 15 min at room temperature. The enzymatic reaction was visualized by the color change and was stopped by the addition of 100 μl of 0.5 M phosphoric acid (H_2_PO_3_). The intensity of color is inversely proportional to the concentration of cortisol in the samples. Absorbance was observed at 450 nm on a microtiter plate reader (spectrophotometer) within 10 min of stop solution addition.

Lysozyme activity was measured using the colorimetric method following the standard protocol (44). In a suitable cuvette, 3 ml of *Micrococcus leutus* (Bangalore Geni, India) suspension in phosphate buffer (OD_450_ = 0.5–0.7) was taken, to which 50 μl of diluted serum sample was added. The content of cuvette was mixed well for 15 s and measured using a spectrophotometer at 450 nm. The reading of lysis of the bacteria was immediately recorded at intervals of 15, 30, and 270 s. A unit of lysozyme activity was defined as the amount of sample causing a reduction in absorbance of 0.001 per minute and lysozyme activity is expressed as U/min.

The immunoglobulin M (IgM) in fish serum was measured using a commercial ELISA kit following the manufacturer’s instruction (Bioassay Technology Laboratory, China). In brief, 50 μl of the standard sample was added to a standard well containing biotinylated antibody. Afterwards, 40 μl of sample, 10 μl of anti-COR antibody, and 50 μl streptavidin-HRP were added into wells. The solution was thoroughly mixed and covered with a plate sealer. The plate was incubated for 60 min at 37°C. Later, the cover was removed and washed five times with wash buffer. During each washing, minimum 350 μl of wash buffer was kept for 30 s to 1 min. Fifty microliters of substrate solution A and 50 μl of substrate solution B were added into wells. The plates were sealed and inculated for 10 min at 37°C under dark conditions. Afterwards, 50 μl of stop solution was added in each well, and a color change from blue to yellow color was observed. The OD was measured immediately within 10 min after the addition of a stop solution using a microplate reader at 450 nm.

### RNA extraction and reverse transcription

The total RNA was extracted using Trizol^®^ reagent following the manufacturer’s instructions. In brief, 0.1 g of each liver tissue sample was weighed and homogenized aseptically for 15–30 s at room temperature with 1 ml of chilled Trizol^®^. The homogenate was incubated for 5 min at 20°C. Then, 200 μl of chloroform was added to the homogenate, mixed vigorously for 15 min at 20°C and centrifuged at 10,000 rpm for 10 min. The upper aqueous layer was transferred to a fresh tube and 500 μl of isopropanol was added to it. The mixture was then kept at −20°C for 2 h and centrifuged again at 10,000 rpm for 10 min. The pellet obtained was washed with 75% ethanol, centrifuged at 7,000 rpm for 10 min, and air-dried to remove the traces of ethanol. The RNA pellets were dissolved in 50 μl of sterile DEPC-treated water and stored at −20°C until further use. The RNA was then treated with DNase I (RNase free; Thermo Scientific, India) to remove the genomic DNA contamination, and its concentration (in ng/µl) and quality were obtained at absorbance 260/280 using a NanoDrop spectrophotometer (Thermo Scientific, India). The integrity of RNA was checked on 2% agarose gel.

Subsequently, reverse transcription was done with the RevertAid™ H Minus First Strand cDNA Synthesis Kit (Thermo Fisher Scientific, India) according to the manufacturer’s guidelines. Briefly, 1 µg of total RNA and 1 µl of random hexamer primer solution was mixed first. Then, 8 µl of reaction mixture containing 4 µl of 5× reaction buffer (0.25 mol^−1^ Tris-HCl, pH 8.3, 0.25 mol^−1^ MgCl_2_, and 0.05 mol^−1^ DTT), 2 µl of 0.01 mol^−1^ dNTP mix, 20 units of ribonuclease inhibitor, and 200 units of RevertAid™ H minus M-MuLV reverse transcriptase were added. The reaction mixture was incubated for 5 min at 25°C followed by 60 min at 42°C. The reaction was terminated by heating at 70°C for 5 min and then cooled to 4°C. Complementary deoxyribonucleic acid (cDNA) samples were checked by PCR and stored at −20°C for further use.

### Quantitative real-time PCR analysis

The expression of C3 (complement component), transferrin (acute phase protein), interleukin 1-β (IL-1β) (pro-inflammatory cytokine), and β-actin (housekeeping gene to check for the integrity of RNA) genes was measured by quantitative real-time PCR (RT-qPCR) with a pair of specific primers using StepOnePlus real-time PCR systems (Applied Biosystems) (**Table S2**) (45–47). The amplification was performed in a total volume of 20 µl, containing 10 µl of 2× Maxima SYBR Green/ROX qPCR Master Mix (Thermo Fisher Scientific), 1 µl of cDNA (50 ng), 8 µl of nuclease free water, and 0.5 µl of each specific primer. Master mixes were prepared for each biological replicate of the sample in triplicate, and RT-qPCR for target and reference genes was performed with a four-step amplification protocol: initial denaturation (10 min at 95°C); 40 cycles of amplification and quantification (15 s at 95°C, 30 s at 60°C, and 30 s at 72°C); melting curve (55–95°C) with a heating rate of 0.10°C/s and a continuous fluorescence measurement; and cooling (4°C). A negative control reaction was included for each primer set by omitting template cDNA. The comparative CT method (2^−ΔΔCt^ method) following Livak and Schmittgen (48) was used to analyze the expression level of the target genes and verified by the Pfaffl relative standard curve method (49). The log-transformed 2^−ΔΔCt^ values were subjected to a *t*-test, and *p*-values lower than 0.05 were considered statistically significant.

### Statistical analysis

The data were arcsin transformed to satisfy homoscedasticity and normality requirements as necessary, and then subjected to one-way analysis of variance (ANOVA) followed by Duncan’s multiple range test using the Statistical Software Package for the Social Sciences version 24.0. *p*-values ≤ 0.001 were considered significant.

## Results

### Co-infection of *P. hypophthalmus* by *I. multifiliis* and *A. hydrophila*

**Figure 1:**
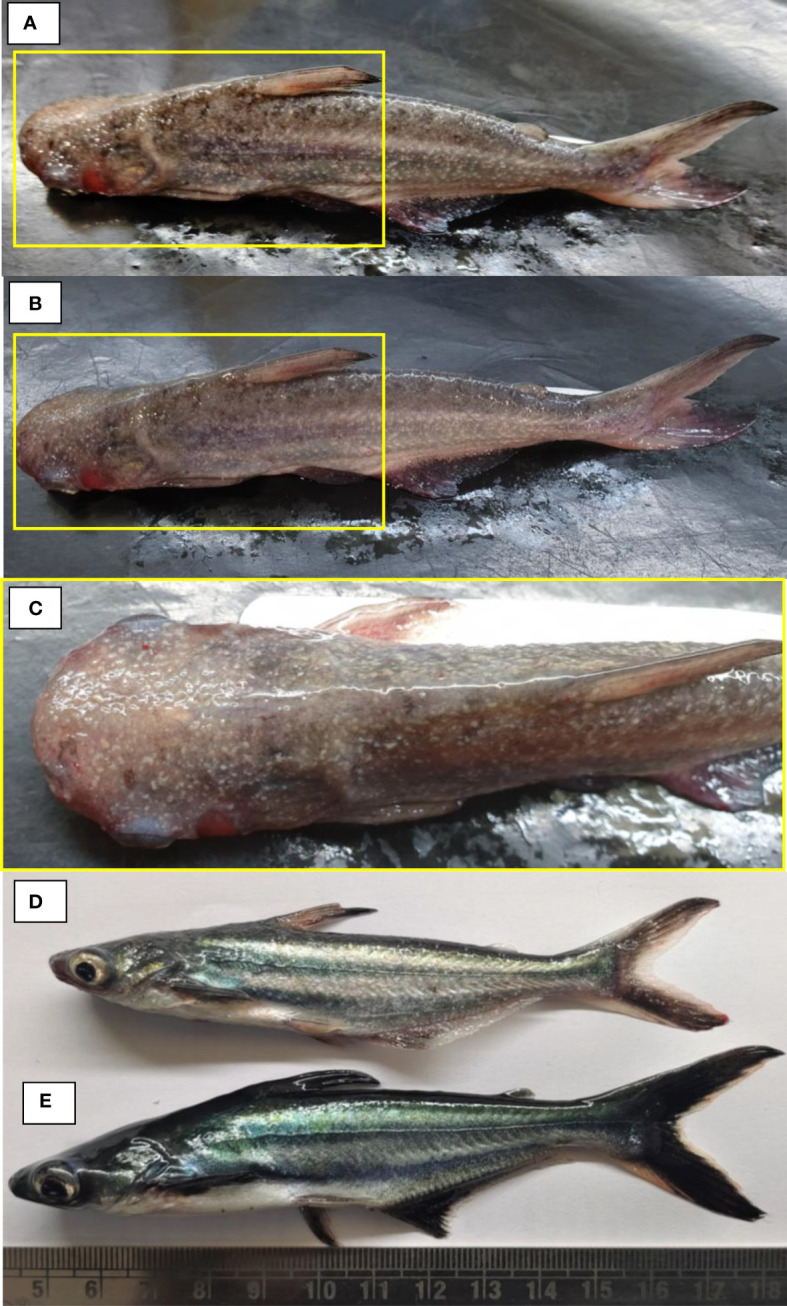
The experimental *Pangasianodon hypophthalmus* used in the study. **(A–D)** Fish showing signs of the disease, including presence of white spot, hemorrhage, ulcer, discoloration, and redness in fins and all over the body surface. **(E)** The control fish appeared healthy, free from possible infection with no external symptoms.

**Figure 2:**
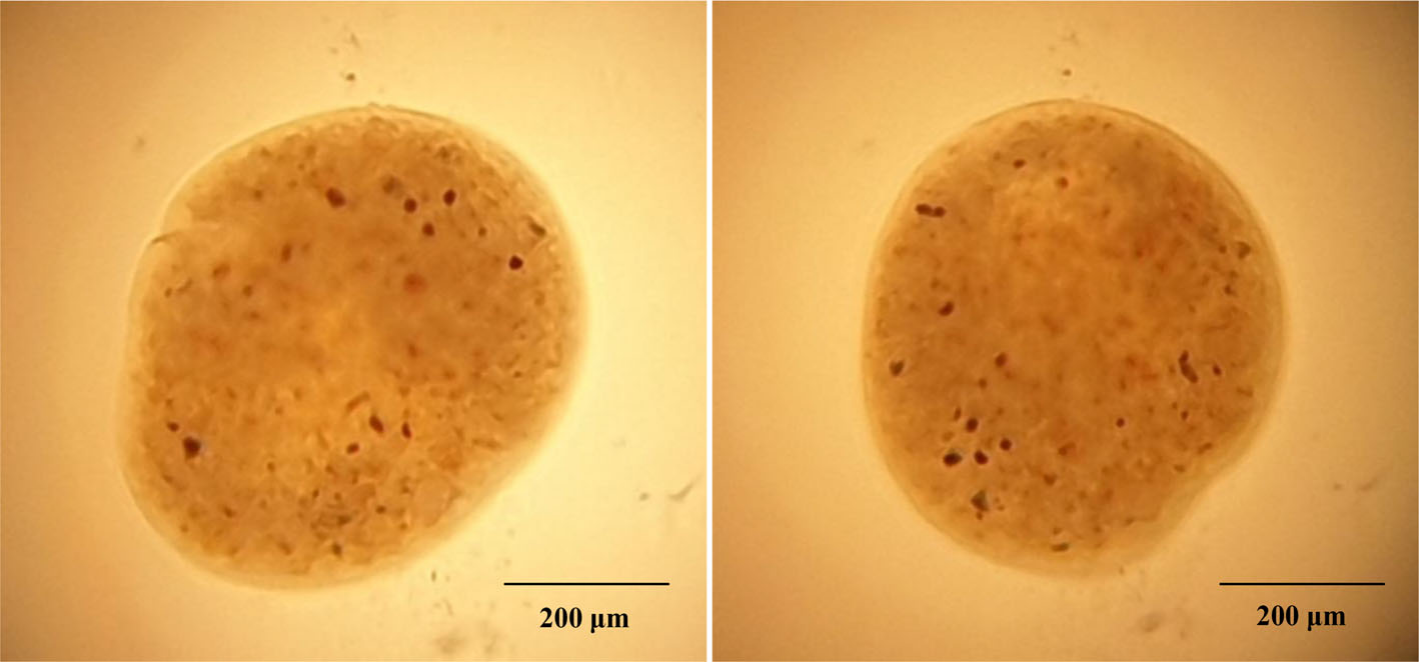
Observation of wet mounts prepared from infected *P. hypophthalmus*. The presence of round to oval shape large ciliated parasite (trophont) indicates the presence of *I. multifiliis*.

**Figure 3:**
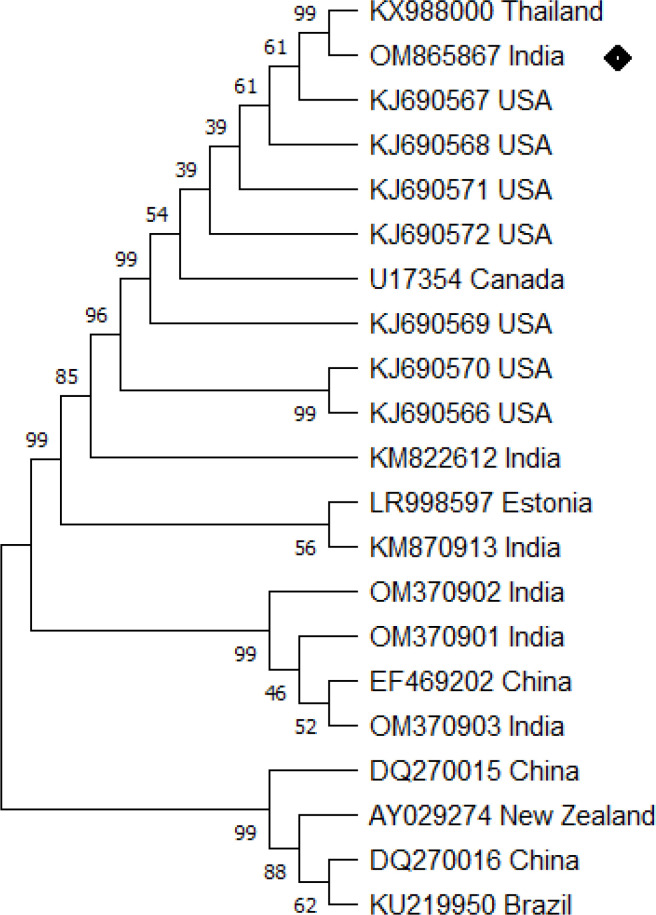
*Ichthyophthirius multifiliis* phylogenetic tree analysis by MEGA X software. The analysis was based on 18S rRNA nucleotide sequences following the Neighbor-Joining method. The *I. multifiliis* (OM865867) isolate identified in the study is indicated by a shaded rectangle.

Initial clinical signs including presence of white spots, hemorrhage, ulcer, and discoloration and redness over the body surface indicate the presence of multiple pathogens in the infected fish (**Figure 1**). Examination of dislodged parasite wet mounts revealed round to oval shape large ciliated parasites (trophont) with horseshoe- or sausage-shaped macronucleus, indicating the pathogenic sign of *I. multifiliis* infection (**Figure 2**). Subsequently, the 18S rRNA gene sequence analysis revealed that the isolated parasitic species was *I. multifiliis*. The sequence has been submitted to GenBank and the Accession number is OM865867. The observed sequences were then subjected to BLAST-N search and results showed that it has 100% similarity with *I. multifiliis* (NCBI GenBank Accession number KJ690572 and KJ690568). The phylogenetic tree prepared from the sequence accessed from NCBI is shown in **Figure 3**.

**Figure 4:**
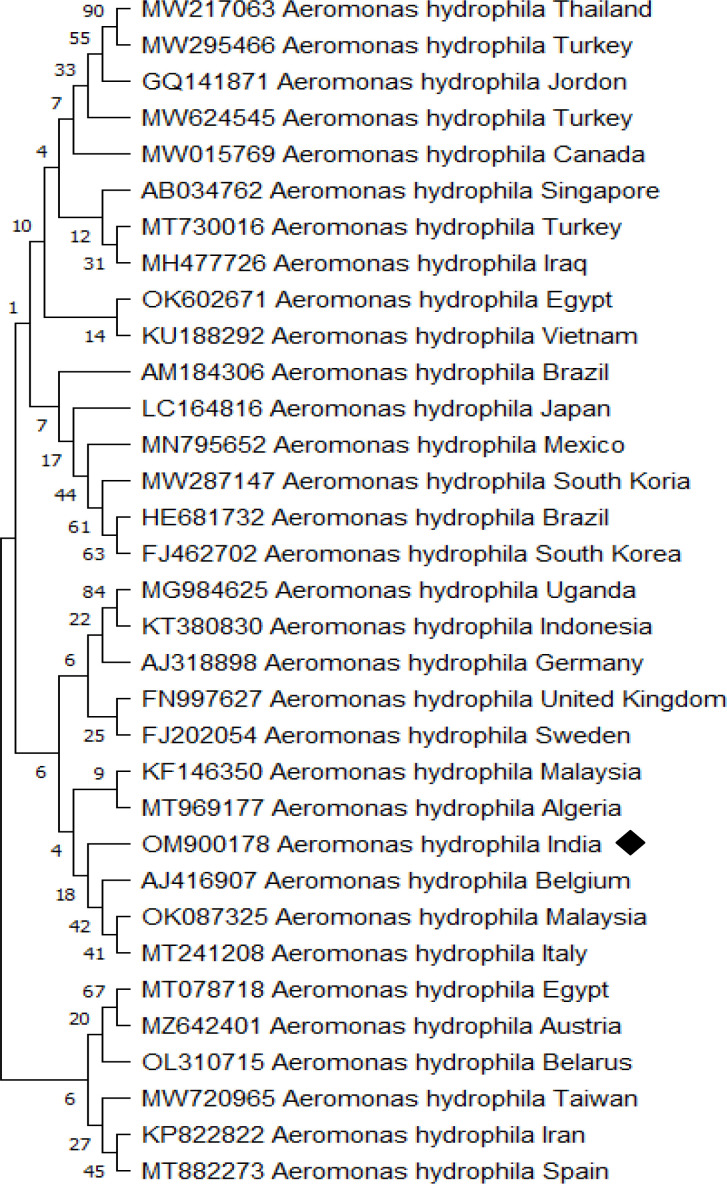
*Aeromonas hydrophila* phylogenetic tree analysis by MEGA X software. The analysis was based on 16S rRNA nucleotide sequences following the Neighbor-Joining method. The *A. hydrophila* (OM900178) isolate identified in the study is indicated by a shaded rectangle.

Apart from white spots, *P. hypophthalmus* exhibited hemorrhagic lesion and redness over the body surface, which indicates the possible involvement of bacterial pathogen in mortality. The bacterial strain observed from the culture technique was further subjected to 16S rRNA gene and phylogenetic analysis. The amplified 16S rRNA gene from the strain was sequenced and submitted to GenBank and accession number OM900178 was obtained. The BLAST analysis against the non-redundant database showed 99.22% identity with *A. hydrophila* (GenBank Accession Number: MT384379 and CP046954). Afterwards, a phylogenetic tree was prepared with the sequence accessed from NCBI (**Figure 4**). The bacterial strain isolated from fish samples exhibited properties of Gram-negative upon gram staining. The biochemical test showed that strain was mostly positive for H_2_S production, citrate, catalase, glucose hemolysis, indole, sucrose, lactose, oxidase, arabinose, mannitol, and VP (Voges Proskauer). Moreover, the strain displayed negative results for urease, arabitol, sorbitol, and xylose activity. Taken together, the molecular sequence and biochemical analysis revealed that the isolated bacterial strain was *A. hydrophila*.

### Co-infections induce histopathological changes

**Figure 5:**
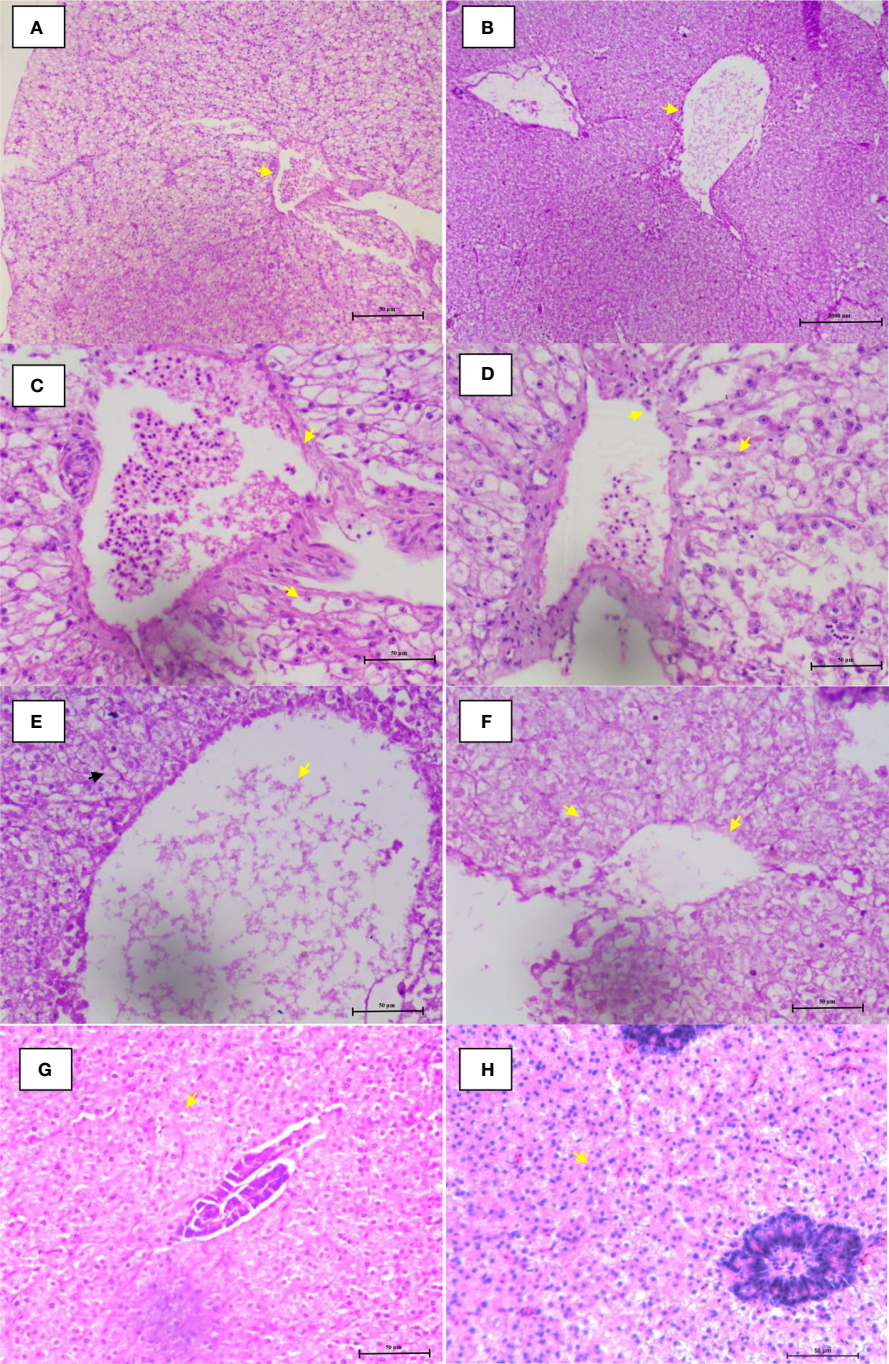
Microphotographs of histological section (H&E) of liver during co-infection. **(A, B)** Lymphocytes migrated from blood vessel and central vein were engorged with blood cells. **(C–F)** Hemorrhage, nuclear pyknosis, degenerated nuclei, blood congestion, degenerated hepatocytes, and vacuolation were observed. **(G, H)** Control group fish liver tissue appearing normal with intact hepatocytes. The arrowhead in figures **(A–H)** represents the cellular changes associated with co-infection in P. hypophthalmus.

The histological analysis demonstrates that co-infection of *I. multifiliis* and *A. hydrophila* induces varying degrees of cellular changes in *P. hypophthalmus*. The results showed that liver tissue was moderately swollen near the margin of the central vein during infection. The tissue showed dilation of the central vein engorged with blood cells with loss of nucleus in the hepatocyte (**Figures 5A, B**). Moderate necrosis near the margin of the central vein was also observed (**Figures 5C–F**). Moreover the control group showed normal histological detail of the liver tissue with intact hepatocytes, appearing normal (**Figures 5G, H**).

### Isolated *A. hydrophila* strain displays high virulence

We next sought to investigate the virulence of the isolated *A. hydrophila* strain possibly involved in the disease development and mortality of *P. hypophthalmus* during co-infection. The cumulative mortality rate of *P. hypophthalmus* post-challenge with *A. hydrophila* is presented in **Table 2**. The control fish did not have mortality or clinical sign of disease during the experimental challenge. However, the fish challenged by intraperitoneal injection with bacterial suspension mostly developed subcutaneous hemorrhagic ulcers of about 0.7–1.8 cm diameter. Reddening at the injection sites and ulcer on the mouth region were also observed. Later, the bacterium was re-isolated from the liver, blood, and kidney of challenged fish and reconfirmed as *A. hydrophila*.

**Table 2:** Survival assay of *Pangasianodon hypophthalmus* challenged with *Aeromonas hydrophila*.

Bacterial species	Survival % (mean ± SE)				
	24 h	48 h	72 h	96 h	120 h
*Aeromonas hydrophila* (10^7^ CFU/ml)	10 ± 1.2	0 ± 0	0 ± 0	0 ± 0	0 ± 0

Among several challenge doses (10^2^, 10^3^, 10^4^, 10^5^, 10^6^, and 10^7^ CFU/ml), the *P. hypophthalmus* injected with 10^7^ CFU/ml showed 100% mortality within 48 h post-injection. Survival was recorded after every 24 h until 120 h and values are representative of mean ± SE.

The hemolytic proteins from the pathogenic *A. hydrophila* strain are one of the important bacterial virulence factors that function by assembling identical subunits into a membrane-spanning pore (50, 51). In parallel with survival assay, results showed that the *A. hydrophila* strain exhibited significant higher hemolytic activity in blood agar (**Table 3**). The results indicate that the *A. hydrophila* strain isolated from *P. hypophthalmus* might be involved in the co-infection mechanism resulting in the high mortality rate of fish.

**Table 3:** Hemolysin assay of *Aeromonas hydrophila* isolated from diseased *Pangasianodon hypophthalmus*.

Bacterial species	Ratio clear zone and colony diameter (mm)
*Aeromonas hydrophila*	3.6 ± 0.08

The strains exhibited hemolytic activity on tryptone soya broth (TSB) supplemented with 5% defibrinated sheep blood (results are expressed as mean ± SE).

### *C. longa* (turmeric) oil protects *P. hypophthalmus* against co-infection

**Figure 6:**
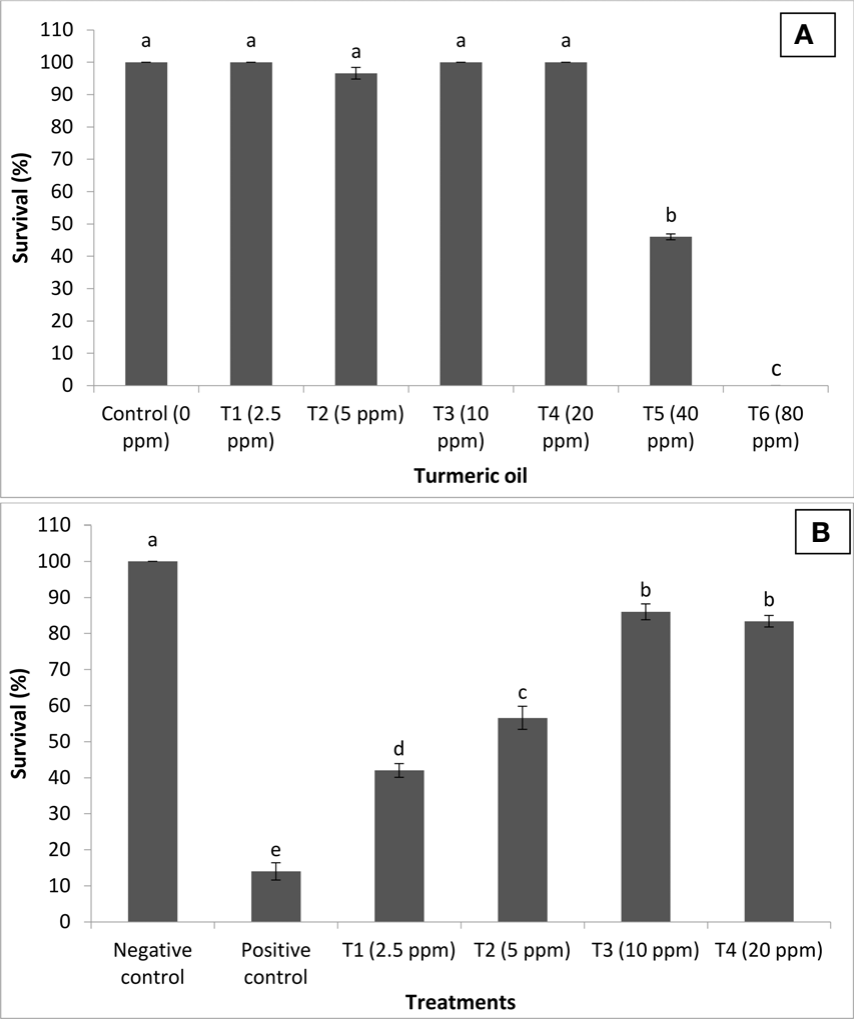
*Curcuma longa* (turmeric) oil treatment protects *Pangasianodon hypophthalmus* fingerlings against *I. multifiliis* and *A. hydrophila* co-infection. **(A)** Toxicity of turmeric oil to *P. hypophthalmus* fingerlings. The fingerlings were treated with oil extract at the indicated doses for 120 h. The non-treated fingerlings served as control. Survival was recorded 120 h post-treatment. Error bars represent the standard error of five replicates; different letters indicate significant differences (*p* < 0.001). **(B)** Survival (%) of turmeric oil-treated *P. hypophthalmus* fingerlings after 120 h of co-infection. The fingerlings were treated with oil extract at the indicated doses in the *I. multifiliis* and *A. hydrophila* co-infected *P. hypophthalmus*. Non-pretreated fingerlings that were either co-infected (positive control) or not (negative control) served as controls. The error bars represent the standard error of five replicates; different letters indicate significant differences between treatment groups (*p* < 0.001).

In the next experiment, the efficacy of turmeric oil in inducing resistance against *I. multifiliis* and *A. hydrophila* co-infection was investigated. The essential oil yield was recorded as 2.65% (V/W). The oil was found rich in bioactive sesquiterpenoids such as ar-turmerone (44.83%), curlone (20.59%), and turmerone (8.29%). The ar-curcumene (2.43%), alpha-zingiberene (1.62%), beta-sesquiphellandrene (2.20%), 1-methyl adamantane (2.84%), alpha-phellandrene (0.76%), and alpha-longipinene (0.68%) were the other bioactive molecules present significantly contributing to the antimicrobial efficacy of the essential oil. The fingerlings exposed to turmeric oil in the range of 2.5 to 20 ppm (T1–T4) did not exhibit any significant difference in survival when compared with unexposed, control fingerlings (**Figure 6A**). In contrast, 40 and 80 ppm (T5 and T6) oil treatment significantly induces high toxicity, and 60%–100% moratlity was observed in the group. Interestingly, the turmeric oil treatment had no effect on fish growth and hepatosomatic index in the tested concentration. This result indicates that turmeric oil appeared to be toxic at high concentration under the present experimental conditions.

We next investigated whether turmeric oil could confer protection to the host *P. hypophthalmus* fingerlings against co-infection. We found that *P. hypophthalmus* fingerlings that received turmeric oil treatment in the range of 2.5 to 20 ppm (T1–T4) exhibited a significant increase in the survival as compared to positive control. However, the maximum survival (~2-fold) was observed at concentrations between 10 and 20 ppm (T3–T4) (**Figure 6B**). Taken together, the results showed that turmeric oil plays an essential role in protecting *P. hypophthalmus* fingerlings against *I. multifiliis* and *A. hydrophila* co-infection.

### *C. longa* (turmeric) oil reduces stress and enhances antioxidant defense

**Figure 7:**
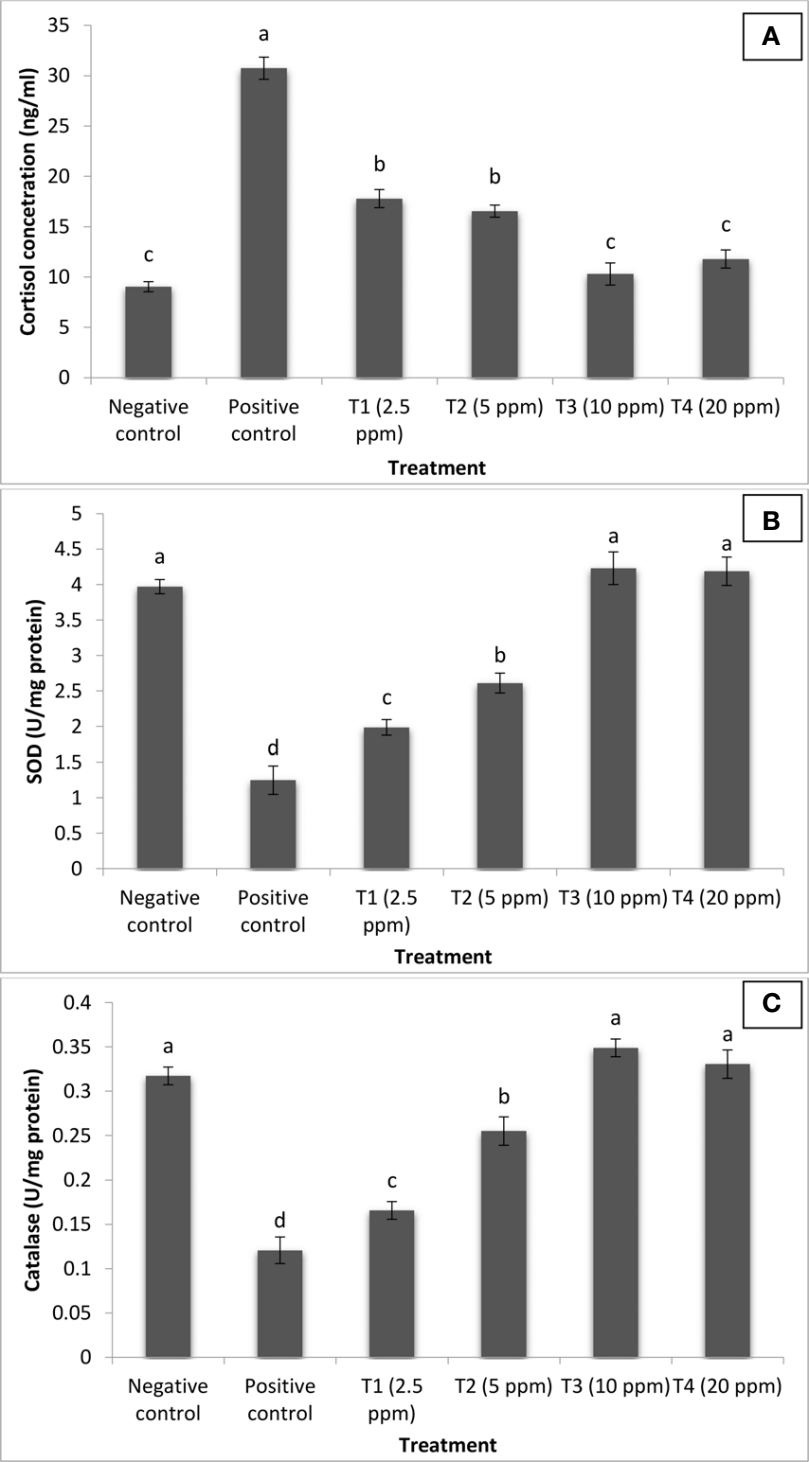
*Curcuma longa* (turmeric) oil treatment reduces stress and enhances antioxidant defense of *Pangasianodon hypophthalmus* fingerlings against *I. multifiliis* and A. *hydrophila* co-infection. The serum samples of *P. hypophthalmus* fingerlings were collected from six treatment groups. The fingerlings in T1–T4 groups were treated with oil extract at the indicated doses. Non-pretreated larvae that were either co-infected (positive control) or not (negative control) served as controls. **(A)** Cortisol concentration (ng/ml); **(B)** superoxide dismutase (SOD) activity (U/mg protein); and **(C)** catalase (CAT) activity (U/mg protein). Error bars represent the standard error of five replicates; different letters indicate significant differences between treatment groups (*p* < 0.001).

We next sought to investigate the mechanism of action of turmeric oil in inducing resistance against co-infection. The findings revealed that cortisol concentration was at basal or resting level in the negative control group (NC) of fingerlings. Moreover, significant positive correlations between *I. multifiliis* and *A. hydrophila* co-infection and cortisol level were observed in *P. hypophthalmus*. The positive control group (PC) of fingerlings has a significantly higher level of cortisol in the serum. Interestingly, the turmeric oil treatment significantly decreased the cortisol level and a lower value was recorded in the 10 ppm treatment group (**Figure 7A**).

SODs and CAT located within mitochondrial and cytosolic cellular compartments are primary antioxidant defense component in fish responsible for detoxification of toxic superoxide anion radicals. In our study, the activity of SOD and CAT was significantly elevated following turmeric oil treatment. Highest values were recorded in treatment T3 groups supplemneted with 10 ppm turmeric oil (**Figures 7B,C**). Moreover, significantly lower values were reported in the PC group of fingerlings. These results indicate that reduced stress and enhanced antioxidant response generated by turmeric oil appeared to be, at least in part, involved in the induction of protective immunity within the *P. hypophthalmus* fingerlings.

### Supplementation of *C. longa* (turmeric) oil enhances non-specific and specific immune response

**Figure 8:**
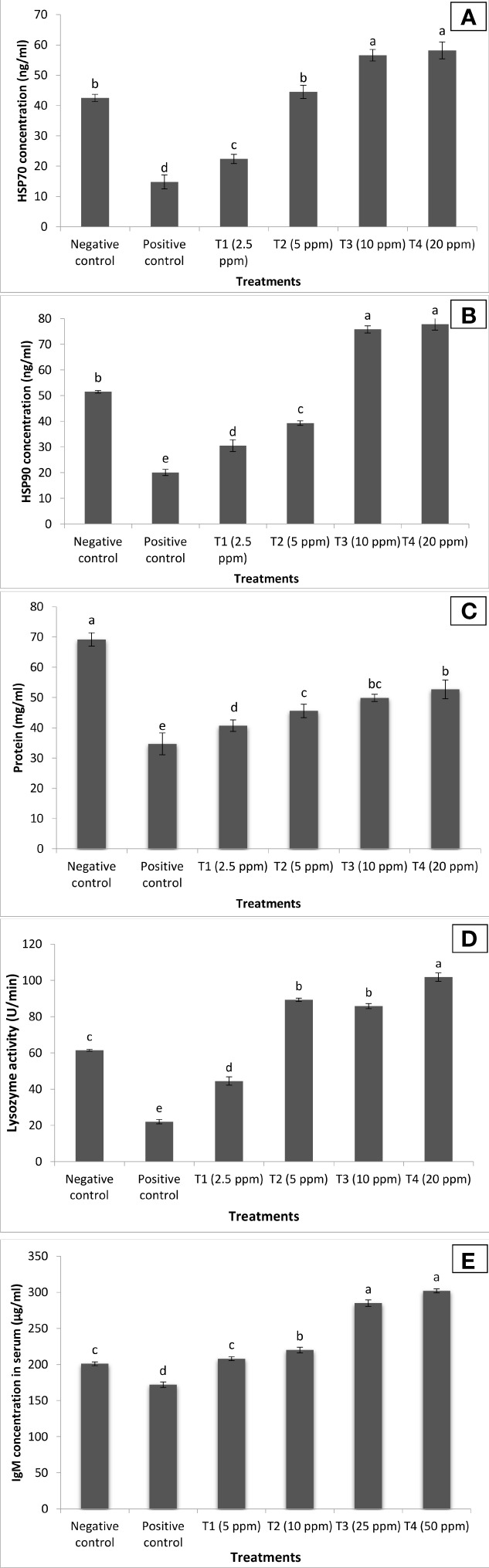
*Curcuma longa* (turmeric) oil treatment enhances both specific and non-specific immune response of *Pangasianodon hypophthalmus* fingerlings against *I. multifiliis* and A. *hydrophila* co-infection. The serum samples of *P. hypophthalmus* fingerlings were collected from six treatment groups. The fingerlings in T1–T4 groups were treated with oil extract at the indicated doses. Non-pretreated larvae that were either co-infected (positive control) or not (negative control) served as controls. **(A)** HSP70 concentration (ng/ml); **(B)** HSP90 concentration (ng/ml); **(C)** protein level (mg/ml); **(D)** lysozyme activity (U/min); and **(E)** IgM concentration (μg/ml). Error bars represent the standard error of five replicates; different letters indicate significant differences between treatment groups (*p* < 0.001).

To gain insight into this, we used enzymatic and molecular assay to assess the role of turmeric oil on non-specific and specific arms of immunity involved in generating resistance in *P. hypophthalmus* fingerlings against co-infection. We found that *P. hypophthalmus* fingerlings treated with turmeric oil showed a significant increase in the HSP70 and HSP90 activity (**Figures 8A, B**). The maximum activity (~2-fold in both HSP70 and HSP70) was observed in T3–T4 treatment groups (10–20 ppm oil concentration). Furthermore, our analysis revealed that turmeric oil treatment to fingerlings showed a significant difference in the total protein level against co-infection (**Figure 8C**). The protein concentration was significantly increased in the treated fingerlings, and highest values were recorded in the T3 group supplemented with 10 ppm turmeric oil. We further examined the secreted immunoglobulins’ concentration possibly induced by turmeric oil. The lysozyme activity increased and differed significantly among the various treatment groups compared to control, and the highest value was found between T2 and T4 (**Figure 8D**). Lysozyme activity in all the treated groups of fish, supplemneted with turmeric oil, showed an increasing trend as compared to control. Treatment group T4 showed the highest lysozyme activity. The IgM activity in the serum of *P. hypophthalmus* fingerlings was significantly upregulated in the treatment group when compared with the negative and positive control fingerlings (**Figure 8E**). In parallel with non-specific immune parameters, higher IgM activity was observed in the T3–T4 groups.

**Figure 9:**
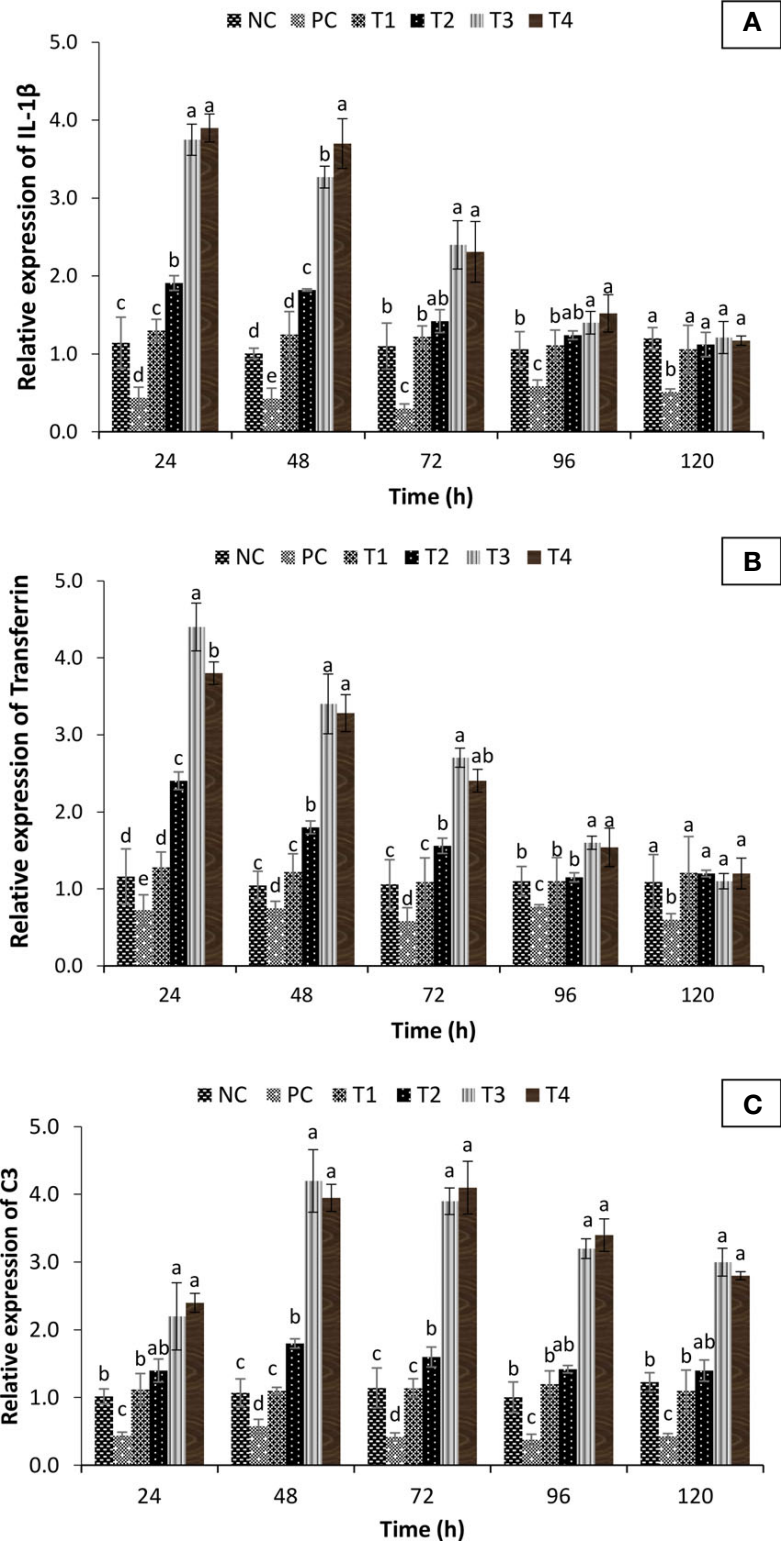
Fold change in immune gene expression of *Pangasianodon hypophthalmus* fingerlings against *I. multifiliis* and *A. hydrophila* co-infection. **(A)** Expression of interleukin-1β (IL-1β). **(B)** Transferrin and **(C)** C3 complement system as determined by quantitative real-time PCR. The expression level in the control group (- co-infection) was regarded as 1.0 and thereby the expression ratio of the positive control group (+ co-infection) and treatments (+ co-infection and turmeric oil) was expressed in relation to the control group. The results are the mean ± SE (*n* = 3) and different letters indicate significant differences between treatment groups (*p* < 0.05).

To further study the transcriptional modifications occurring in the turmeric oil-supplemented group against co-infection, the *in vivo* temporal expression of C3 (involved in both innate and adaptive immune response), transferrin (acute phase protein involved in innate immune response), and interleukin 1β genes (IL-1β) (pro-inflammatory cytokine) was investigated and compared with positive and negative control groups. The qPCR analysis showed that interleukin 1β genes (IL-1β), transferrin, and complement component C3 exhibited differential expression profile in turmeric oil treatment groups as compared to the control group. The transcription of pro-inflammatory cytokine, IL-1β, and transferrin (acute phase protein) was significantly upregulated 24 h post-treatment in T3 and T4 groups (**Figures 9A, B**). Moreover, the expression of C3, the central component of the complement system, was significantly upregulated 48 h post-treatment and maximum values were recorded (fourfold or more) in T3 and T4 groups (**Figure 9C**). Taken together, these results imply that *C. longa* (turmeric) oil enhances both non-specific and specific immunity, which possibly leads to increased survival of *P. hypophthalmus* fingerlings against *I. multifiliis* and *A. hydrophila* co-infection.

## Discussion

Co-infection is a condition where more than one pathogen infects the same host, either simultaneously or as a secondary concurrent infection (52). The interactions between the invading pathogens often alter the host susceptibility and increase the likelihood of disease outbreak through synergistic and, more rarely, antagonistic interactions (53, 54). Although co-infection is very common in aquatic animals, very few attempts have been made to unravel the mechanism of co-infections in fishes to determine host susceptibility, disease course, and severity in order to develop a suitable management approach to control these infections. Hence, there is a need to develop natural compounds/molecules that could confer protection and/or enhance immune reactivity against biotic and abiotic stressors, in a manner conceptually equivalent to probiotics, vaccines, or immunostimulants (40, 55–59). These compounds have received great attention in recent years with regard to fish because of the additional biodegradable and biocompatible properties that work on One Health approach, particularly relevant in food safety and combating antibiotic resistance (16, 60, 61). *C. longa* (turmeric), a medicinal herb that belongs to Zingiberaceae family, is widely distributed and available in southeastern countries including India. This plant imparts several beneficial effects including anti-stress responses (62); enhanced fish growth and antimicrobial activity (63); antioxidant (64), anti-inflammatory (65), and immunostimulatory (66) properties; and stimulation of digestive enzyme secretion (67). This motivated us to investigate the potential role of turmeric oil against a co-infection condition induced by *I. multifiliis* and *A. hydrophila*. The study describes that turmeric oil induces anti-stress, antioxidant, and non-specific and specific immunity that leads to increased survival of *P. hypophthalmus* fingerlings against *I. multifiliis* and *A. hydrophila* co-infection.

In the study, a co-infection of *I. multifiliis* and *A. hydrophila* was reported from *P. hypophthalmus* exhibiting typical clinical signs including the presence of white spots, ulceration, fin erosion, lethargy, and decreased appetite. The clinical signs and observed parasite structure recorded in this study coincide with previous works reported during *I. multifiliis* infection (white spot disease) in fish (68, 69). In addition, the infection induces a varying degree of cellular changes in *P. hypophthalmus* including degenerated hepatocytes, vacuolation, and migration of lymphocyte from blood vessel towards hepatocytes. Since *I. multifiliis* and *A. hydrophila* are two common microbes of the freshwater ecosystem, the co-infection of these two pathogens increased the severity of disease, causing high mortality in *P. hypophthalmus*. Although we could highlight that co-infection increases the severity of disease in fishes, we could not explain how the prior parasite or bacterial infections enhance the ability of the microbes to cause infection and mortality in fishes (70–73).

The ability to stimulate the defense system of vertebrates is considered to be central to develop resistance and control the pathogenesis of microbial pathogens (74, 75). In case of aquatic animals that are constantly exposed to microbial pathogens, non-specific and specific immune factors might be a potential preventive modality to manage both biotic and abiotic stressors in the aquaculture farming system (76, 77). Although few works have shown that plant-based and natural compounds increase the host resistance against bacterial infection by facilitating enhanced immunity (78, 79), the role of these compounds against co-infection, particularly *I. multifiliis* (parasitic) and *A. hydrophila* (bacterial), and the molecular mechanism behind the generation of protective effect in host remain to be established. In this study, using a host–pathogen model system, it was shown that turmeric oil provides protection to *P. hypophthalmus* fingerlings against co-infection.

Stress-mediated impairment of immune function has been widely described in cultured and wild fish, and associated with an increased susceptibility to disease (80). Furthermore, the water temperature also dramatically changes the response of fish to stress and the recovery dynamics (81). The stress response is initiated and controlled by the production of corticosteroids (mainly cortisol, a steroid hormone with many biological activities including gluconeogenesis and immunosuppression). Cortisol, a principal corticosteroid released as part of the primary stress response and normally associated with stressful and disease conditions, plays a critical role in mediating adaptive metabolic, physiological, and behavioral adjustments (82, 83). However, prolonged elevation of cortisol, due to extended or repeated exposure to a stressor, is often associated with adverse health effects (80). The level of cortisol in fish indicates the health status of animals, as extended increased cortisol level is often negatively associated with growth, development, disease resistance, immunity, and reproduction (83). The oxidative stress and infectious disease are interrelated, and the emergence of one phenomenon leads to the development of another and *vice versa*. For instance, the excessive production of reactive oxygen species (ROS) and reactive nitrogen species (RNS) by activated immune cells during microbial disease creates a highly cytotoxic environment that leads to imbalanced immune response and direct damage of target organs (84–86). In parallel, the study showed that co-infection of *I. multifiliis* and *A. hydrophila* significantly induced a stressful condition in *P. hypophthalmus* fingerlings, resulting in higher cortisol and lower antioxidative enzyme activity in the positive control group of fish. Since, during disease outbreak and mortality, low water temperature (~18°C) was found, this might have accelerated the parasitic and bacterial co-infection resulting in high mortality in *P. hypophthalmus*. Moreover, a positive correlation between cortisol concentrations in serum and mucus secretion was observed in *P. hypophthalmus* with co-infection conditions (87). This may be explained by the fact that *I. multifiliis*, a ciliated parasite, which parasitizes the epithelial surface of fish, induces the production and mucus in the skin surfaces (88). Interestingly, supplementation of turmeric oil exhibited a protective effect in *P. hypophthalmus* fingerlings against co-infection by the possible mechanism of anti-stress and antioxidant activity. In the turmeric oil treatment groups, reduced cortisol and enhanced antioxidant enzyme activity (SOD and CAT) were observed. The antioxidant enzymes are responsible for the generation of antioxidant defense response and imparting protection from oxidative stress by detoxification of free radicals (89). These results were consistent with the known beneficial roles of plant-based compound to induce resistance in host and prevent the microbial infection functionally through anti-stress, antioxidant, and immunostimulatory properties (90, 91).

The plant-based compound and its derivatives mediated improved resistance against microbial pathogens and have been linked to enhanced immune response in fishes (35, 39, 56). Additionally, there exists a correlation between the reduced stress and enhanced antioxidative response with non-specific (HSP70, HSP90, lysozyme) and specific immune response (IgM) of animals against microbial infection (39, 40, 92). Interestingly, turmeric oil significantly enhances HSP70, HSP90, lysozyme, and IgM level in *P. hypophthalmus* fingerlings against *I. multifiliis* and *A. hydrophila* co-infection. Our results indicate that increased survival in *P. hypophthalmus* treated with turmeric oil is most likely due to a significant improvement in the health status of the fish. This is due to the fact that apart from reducing cortisol level and enhancing antioxidative response, turmeric significantly induces both non-specific and specific immunity of *P. hypophthalmus* fingerlings.

The fish immune response is a cascade of diverse reactions that aims to eliminate the recognized foreign agent and restore the homeostasis (93). The expression of immune genes (e.g., interleukin 1β, transferrin, and C3 complement system) is usually considered as a sign for immune stimulation or enhanced immune response (92, 94). Interleukin-1β (IL-1β), a classic pro-inflammatory cytokine, plays a central role in early inflammatory response by mediating immediate and vigorous response, inducing a number of inflammatory reactions (95). Transferrins are physiologically important multi-task globular proteins that play an important role in binding and transport of iron, elucidating antimicrobial activity and executing a vital role in growth, differentiation, and cyto-protection processes (96). Serum transferrin concentration in vertebrates varies with the infectivity of the microorganism, closely reflecting stress conditions or infection and hence considered as an important biomarker for acute phase response (97). Additionally, the C3 is the central component of the complement system, involved in both innate and adaptive immune defense and has many functions including opsonization, direct killing, regulation of the immune response, and mediation of inflammation (98). Research on fish showed upregulation of C3 expression in the liver following infection with the parasite *I. multifiliis* (*Cyprinus carpio*) and bacteria *Yersina ruckeri* (rainbow trout) (99, 100). In the present study, significant upregulation of IL-1β, transferrin, and C3 gene expression was observed post-turmeric oil treatment in *P. hypophthalmus* with co-infection conditions. The transcription of IL-1β and transferrin genes was significantly enhanced at 24 h post-treatment while the highest expression of C3 gene was observed at 48 h post-treatment. The gene expression analysis corroborated that essential oil-mediated immunomodulatory properties play an essential role in providing protection in *P. hypophthalmus* fingerlings against *I. multifiliis* and *A. hydrophila* co-infection.

In conclusion, the study was designed to investigate the possible co-infection condition, isolate the causative agent, and develop a suitable management strategy to control the outbreak and mortality in *P. hypophthalmus*. We reported that *I. multifiliis* (parasitic) and *A. hydrophila* (bacterial) were involved in the co-infection in *P. hypophthalmus*. Furthermore, *C. longa* (turmeric) oil was used and investigated for potential immunomodulatory properties against co-infection in fingerlings. Our study provides strong evidence that initial generation of anti-stress, antioxidative response and non-specific and specific immunity play a key role in the protection of *P. hypophthalmus* fingerlings against co-infection. However, in light of our findings, it is questionable whether the isolated microbial pathogens are primary or secondary pathogens, as virulence and interaction of two pathogens were not estimated. Nevertheless, the ability of turmeric oil to boost immunity and enhance resistance in *P. hypophthalmus* makes it a potent biocontrol agent that may be valuable to treat co-infection condition in other fish species. Although we could not investigate the antiparasitic and antibacterial role of turmeric oil, the results obtained here add new information on turmeric oil immunomodulating properties and advance the knowledge of this compound as a potential disease-mitigating agent.

## Data availability statement

The datasets presented in this study can be found in online repositories. The name of the repository and accession number can be found below: NCBI GenBank; OM900178 and OM865867.

## Ethics statement

This study was reviewed and approved by Organization for Economic Cooperation and Development (OECD) guidelines were followed for the handling and care of experimental animals. The animal utilization protocol was approved by Institutional Animal Ethics Committee, ICAR-Central Inland Fisheries Research Institute, Kolkata, India, (IAEC/2021/04) for the experimental setup.

## Author contributions

VK and BD designed the study. VK and HS collected samples. VK, SR, SP, SD, RD, and AJ made the analysis. VK prepared the manuscript. BD, HC, AB, and BB reviewed and edited the final version. All authors contributed to the article and approved the submitted version.

## Acknowledgments

We would like to acknowledge the technical assistance provided by Mr. Kampan Biswas, Mr. Jayanta Pramanik, Mr. Aurobinda Upadhyay, and Mr. Abhijit Pakhira. The authors are thankful to Director ICAR-Central Inland Fisheries Research Institute (ICAR-CIFRI) and other technical and supporting staff for the financial and technical support.

## Conflict of interest

The authors declare that the research was conducted in the absence of any commercial or financial relationships that could be construed as a potential conflict of interest.

## Publisher’s note

All claims expressed in this article are solely those of the authors and do not necessarily represent those of their affiliated organizations, or those of the publisher, the editors and the reviewers. Any product that may be evaluated in this article, or claim that may be made by its manufacturer, is not guaranteed or endorsed by the publisher.
