# Supplementary material for: Outbreak of Ichthyophthirius multifiliis associated with Aeromonas hydrophila in Pangasianodon hypophthalmus: The role of turmeric oil in enhancing immunity and inducing resistance against co-infection
Source: Front Immunol. 2022 Sep 2;13:956478. doi: 10.3389/fimmu.2022.956478 (PMC9478419; doi:10.3389/fimmu.2022.956478)
Supplement: Supplementary file 1 [file Table_1.docx]

Table S1 Chemical composition of *Curcuma longa* (turmeric) essential oil

| Sl. No. | Compound | Molecular formula | Chemical type | Retention time (min) | Concentration (%) |
| --- | --- | --- | --- | --- | --- |
|  | beta-Caryophyllene | C_15_H_24_ | Bicyclic [sesquiterpene](https://en.wikipedia.org/wiki/Sesquiterpene) | 19.437 | 0.27 |
|  | ar-Curcumene | C_15_H_22_ | Mono cyclic sesquiterpene | 21.378 | 2.43 |
|  | alpha-Zingiberene | C_15_H_24_ | Mono cyclic sesquiterpene | 21.740 | 1.62 |
|  | beta-Bisabolene | C_15_H_24_ | Mono cyclic sesquiterpene | 22.135 | 0.38 |
|  | beta-Sesquiphellandrene | C_15_H_24_ | Mono cyclic sesquiterpene | 22.593 | 2.20 |
|  | 2-Hepten-4-one | C_7_H_12_O | Aliphatic ketone | 24.164 | 0.29 |
|  | unidentified | - | - | 24.279 | 1.07 |
|  | Di-epi-alpha-cedrene-(I) | C_15_H_24_ | Cedrane sesquiterpene | 24.546 | 0.22 |
|  | (+)-beta-Atlantone | C_15_H_22_O | Monocyclic sesquiterpenoid | 24.622 | 0.21 |
|  | unidentified | - | - | 24.985 | 1.53 |
|  | unidentified | - | - | 25.093 | 0.36 |
|  | Farnesol 2 | C_15_H_26_O | Farnesane sesquiterpenoid | 25.214 | 0.58 |
|  | unidentified | - | - | 25.608 | 0.97 |
|  | alpha-Longipinene | C_15_H_24_ | Tricyclic sesquiterpene | 25.697 | 0.68 |
|  | alpha-Tumerone | C_15_H_22_O | Monocyclic sesquiterpenoid | 25.863 | 1.18 |
|  | unidentified | - | - | 26.181 | 0.43 |
|  | Ar-tumerone | C_15_H_20_O | Monocyclic sesquiterpenoid (aromatic) | 26.919 | 44.83 |
|  | beta-Tumerone | C_15_H_22_O | Monocyclic sesquiterpenoid | 26.970 | 7.11 |
|  | unidentified | - | - | 27.409 | 0.83 |
|  | Curlone | C_15_H_22_O | Monocyclic sesquiterpenoid | 27.803 | 20.59 |
|  | unidentified | - | - | 28.051 | 0.33 |
|  | (S)-(+)-Curcuphenol | C_15_H_22_O | Monocyclic sesquiterpenoid phenol | 28.147 | 0.38 |
|  | (6S)-2,10-Bisaboladien-1-one | - | - | 28.866 | 1.08 |
|  | alpha Phellandrene | C_10_H_16_ | Monocyclic monoterpene | 29.127 | 0.76 |
|  | unidentified | - | - | 29.336 | 0.62 |
|  | unidentified | - | - | 29.464 | 1.29 |
|  | Adamantane,1-methyl | C_11_H_18_ | Adamantane | 29.616 | 2.84 |
|  | unidentified | - | - | 30.068 | 0.56 |
|  | unidentified | - | - | 30.265 | 0.94 |
|  | Methyl 2-Cyclopropylacetate | C_6_H_10_O_2_ | Cyclopropylacetic acid ester | 30.564 | 1.16 |
|  | unidentified | - | - | 32.454 | 1.58 |
|  | unidentified | - | - | 32.906 | 0.68 |
|  | Identified | | | | 88.81 |
|  | Unidentified | | | | 11.19 |
