# Supplementary material for: Outbreak of Ichthyophthirius multifiliis associated with Aeromonas hydrophila in Pangasianodon hypophthalmus: The role of turmeric oil in enhancing immunity and inducing resistance against co-infection
Source: Front Immunol. 2022 Sep 2;13:956478. doi: 10.3389/fimmu.2022.956478 (PMC9478419; doi:10.3389/fimmu.2022.956478)
Supplement: Supplementary file 2 [file Table_2.docx]

Table S2. List of primers used in the immune gene expression analysis of *P. hypophthalmus*

| Gene name | Primer sequence (5′→3′) | Length | References |
| --- | --- | --- | --- |
| C3  (Complement) | FWD: TCCACCAGAGCCATCCCATA  REV: CACAACTTGAACGCCACCAG | 198 | Sirimanapong et al., 2015; Hoque et al., 2020 |
| Transferrin  (Acute phase response ) | FWD: CACCCCATAACCTTCACCCC  REV: CGCAGTTTTCCCCAAACCAG | 149 |  |
| Interleukin-1β  (pro-inflammatory cytokine) | FWD: CAGAGGCTGAAGCACACTCA  REV: CCTTGTCCTGCCTGCTGTAA | 148 |  |
| β -actin (Housekeeping reference gene) | FWD: ATTGATGCCCCTGGACACAG  REV: GGGTCTGTCCGTTCTTGGAG | 133 |  |
