# Supplementary material for: Outbreak of Ichthyophthirius multifiliis associated with Aeromonas hydrophila in Pangasianodon hypophthalmus: The role of turmeric oil in enhancing immunity and inducing resistance against co-infection
Source: Front Immunol. 2022 Sep 2;13:956478. doi: 10.3389/fimmu.2022.956478 (PMC9478419; doi:10.3389/fimmu.2022.956478)
Supplement: Supplementary file 3 [file DataSheet_1.docx]

Figure S1 GC-MS chromatogram of *Curcuma longa* (turmeric) essential oil
